# School academic climate and oral health (tooth loss) in adolescents

**DOI:** 10.1371/journal.pone.0233505

**Published:** 2020-05-21

**Authors:** Carolina da Franca Bandeira Ferreira Santos, Fabiana Godoy, Valdenice Aparecida Menezes, Viviane Colares, Patrícia Maria Zarzar, Raquel Conceição Ferreira, Ichiro Kawachi

**Affiliations:** 1 School of Dentistry, Graduate Program in Hebiatrics, University of Pernambuco, Recife, PE, Brazil; 2 Department of Paediatric Dentistry, School of Dentistry, Federal University of Minas Gerais, Belo Horizonte, Minas Gerais, Brazil; 3 Department of Social and Preventive Dentistry, School of Dentistry, Federal University of Minas Gerais, Belo Horizonte, Minas Gerais, Brazil; 4 Department of Social and Behavioral Sciences, Harvard T.H. Chan School of Public Health, Boston, MA, United States of America; Ohio State University, UNITED STATES

## Abstract

**Background:**

Preventing tooth loss depends on oral health maintenance behaviors. This study hypothesized that adolescents with educational aspirations have greater motivation to invest in the future, including maintenance of oral health status.

**Aim:**

To analyze the association between a school academic climate of educational aspirations and tooth loss (first permanent molars) among adolescents.

**Methods:**

A cross-sectional study was designed to include 2,500 adolescents (aged 14–19 years) enrolled in public high schools of Olinda located in Northeast Brazil. Multilevel Poisson regression random intercept models were conducted with tooth loss (first permanent molars) as the outcome. The primary cohort of interest was school academic climate, as measured by the proportion of students taking the national high school exams.

**Results:**

Tooth loss of the first permanent molars (assessed by clinical exam) was more prevalent in adolescents from more disadvantaged backgrounds (receiving family allowance, low maternal education). However, after controlling for a wide range of individual characteristics, adolescents enrolled in schools with lower academic climate had a higher prevalence of tooth loss (PR 1.42, 95%CI: 1.09,1.85).

**Conclusion:**

The school academic climate is associated with tooth loss, suggesting that educational aspirations are linked to adolescent oral health maintenance behaviors.

## Introduction

Prevalence of tooth loss increases gradually with age [1] and in adolescents is associated with a number of deleterious outcomes including poor self-esteem [2], low quality of life [3], impact on daily performance [4], as well as impacts on social relations, and labor market outcomes (employability) [5]. It is worth noting that tooth loss before age 35 may be a risk factor for Alzheimer’s disease at 65 years or over [6].

In adolescence, tooth loss often affects the first permanent molars since these are the earliest teeth to erupt [7] and the most affected by caries [8]. Preventing tooth loss depends on oral health maintenance behaviors, e.g., brushing with fluoridated toothpaste to prevent caries, avoidance of cigarette smoking and high consumption of sugars and refined carbohydrates, as well as regular and preventive dental check-ups. Socioeconomic disparities in oral health maintenance behaviors (and oral health status) have been extensively documented [9–12]. Adolescents from disadvantaged backgrounds (income poverty, low parental education) are less likely to engage in preventive behaviors. Socioeconomic inequalities in the practice of oral health maintenance behaviors can be explained by different behavioral theories which focus on disparities in health literacy, time & resource scarcity, differences in norms & attitudes, and self-efficacy [13]. Behavioral theories such as the Theory of Planned Behavior and the Health Belief Model tend to emphasize factors operating at the level of individual decision-making, such as self-control beliefs, attitudes toward the behavior, as well as perceived social norms surrounding the behavior [14, 15]. While these theories provide a powerful set of tools to explain (and intervene on) health behaviors, they also miss the contextual influences that potentially drive behavior change [16]. That is, an exclusively individual focus on health behavior provides an incomplete picture of the social contextual factors which may motivate (or demotivate) the adoption of preventive practices. To address this gap, the “scarcity hypothesis” from the field of behavioral economics suggests that socioeconomic disadvantage is characterized by uncertainty about the future, and that this can help to explain the lack of future orientation among youth raised in poverty [12]. In other words, the lack of motivation to invest for the future could be explained as a “rational” response to the individual’s pessimistic assessments of the future. If the prospects are slim for future employment, or reaching a ripe old age, how can youth be motivated to invest for the future, by avoiding health-damaging habits, or engaging in health maintenance behaviors?

Emerging evidence corroborates the scarcity hypothesis. For example, in the United States after affirmative action in college admissions was struck down by a Supreme Court ruling, rates of cigarette smoking increased among underrepresented minority adolescents [17, 18]. Education offers one of the surest ways for socioeconomically disadvantaged groups to experience upward mobility. Lee et al. show that children with high educational aspirations are more likely to graduate from high school and also to have higher income at age 30 [19]. Adolescents who aspire to attend college are also more likely to do exercise regularly and to abstain from smoking [20]. In the realm of oral health, studies have reported an association between scholastic performance and the likelihood of visiting the dentist in the last year [21, 22].

School climate refers to the quality and character of school life, and encompasses distinct dimensions including: (i) safety, (ii) teaching and learning (academic climate), (iii) relationships between pupils, teachers and students, and (iv) the institutional environment (e.g., physical layout, size, and material resources of a school) [23]. In the educational literature, positive school climate has been related to positive student outcomes, such as higher academic performance [24], better mental health [25], and less bullying [26]. While an association between adolescents’ future planning and academic achievement were reciprocal [27], a positive association between school performance and healthy dietary habits also was found [28]. Accordingly, in the present study, we sought to test one aspect of school climate, viz., the relation between academic climate of educational aspiration and adolescent oral health (as measured by tooth loss).

On the other hand, the association between educational aspiration and oral health status could be endogenous, i.e. there may be unobserved characteristics of the individual (such as conscientious personality) that predicts both school performance *and* oral health. In the present study we sought to test whether the school academic climate is associated with oral health status, over and above individual characteristics. At the individual level, besides sociodemographic characteristics, clinical variables, lifestyle, self-perception of oral health, and dental anxiety contribute to the explanation of the occurrence of tooth loss. The progression of some clinical variables, such as dental caries and periodontal disease, leads to tooth loss [29]. Self-perception of oral health [30] and dental anxiety are variables related to poor oral health because it could function as a barrier for using oral health services contributing to tooth loss [31]. Accordingly, we included at the individual level the following variables: sociodemographic characteristics (age, sex, mother's level of schooling, receiving a family allowance—proxy of poverty), clinical variables (decayed teeth—DMFT 1, and teeth affected by periodontal disease—CPI 3), and subjective ratings (self-perception of oral health and dental anxiety).

In Brazil, a National High School Examination (ENEM) was introduced at the end of 1998. In 2010 the exam started to be used as a criterion for admission to some public universities and for obtaining scholarships at private universities. High schools are ranked publicly based on student performance–the ENEM league table [32]. However, because the exam is not mandatory for students, only schools with at least 50% participation are ranked in the league table [32, 33].

In the present study, we used the average student participation rates in ENEM as a proxy for the school academic climate. This is a *contextual* variable, i.e., it is a measure of the average educational aspiration “climate” in each school. This study hypothesized that a school climate characterized by higher educational aspirations is associated with lower incidence of adolescent tooth loss. Such schools expose their students to a milieu that instills hope for the future, and hence motivates them to invest in their own health.

## Materials and methods

A cross-sectional study was carried out in Olinda, in the state of Pernambuco, Brazil from February to June 2018 and from August to October. In 2018, Olinda had a population of 390,000 residents.

The parents/guardians of all participants under 18-years-old provided written consent while children also provided written assent. Adolescents over 18-years of age consented by themselves. This research was approved by the Ethics Committee of University of Pernambuco (N. 2.361.780).

In 2018, Olinda had 33 state public high schools. Two schools were not included because they held only night classes. Out of the 31 daytime schools, 26 agreed to take part in the study (rate response 83.9%). All students in the 26 schools were invited to participate, and 2,700 returned the questionnaire surveys (overall response rate 37.2%). Almost all classrooms (90.3%) in the involved schools participated in our data collection.

Four trained and calibrated dentists performed oral examinations on the students, according to the methods and criteria recommended by the WHO [34]. Dental caries (DMFT index), presence of tooth and, periodontal condition (Community Periodontal Index-CPI) were evaluated. Inter-rater reliability between examiners was satisfactory for dental caries and periodontal condition, with kappas ranging from 0.82 to 0.91 and, from 0.76 to 0.85, respectively.

Tooth loss was our dependent variable, defined based on the absence of first permanent molars due to dental caries through the missing component of the DMFT score or due to other reasons.

The survey inquired about sociodemographic characteristics and health behaviors adapted from the U.S. Youth Risk Behavior Survey questionnaire [35]. The questionnaire also included the Dental Anxiety Question (DAQ) [36] and the Self-perception of oral health status that was assessed through a single question (How would you describe your oral health?) with a 5-point Likert scale. Sociodemographic variables included gender, age, mother`s schooling and receipt of family allowance (a social safety net in Brazil for poor families (per-capita monthly below $170 BRL/~$45 USD) and extremely poor families (per-capita monthly below $85 BRL/~$23 USD).

Our contextual variables included the school rate of participation in the National High School Examination (ENEM) using the mean of the rate in 2016 and 2017 available in public database. Z-scores were used to standardize the variance in rate of participation. The type of school was also assessed (regular vs. full-time/partial full-time). In Brazil, most schools operate 4 hours a day, but a few schools run full-time, i.e. 8 hours per day.

The Social Vulnerability Index (SVI) is a measure of neighborhood disadvantage made up of 16 indicators in three domains: urban infrastructure, human capital and, income/labor. The SVI is available for each Human Development Unit (HDU) that is the smallest socioeconomically homogeneous area unit in a city. We obtained the SVI and boundaries for each HDU in the city of Olinda from the Brazilian Institute of Geography and Statistics’ 2010 Demographic Census [37].

Data analysis was carried out using STATA/IC version 15.1. Bivariate analysis was conducted using Pearson’s chi-squared test and T-tests for categorical and numerical variables, respectively. Tooth loss (count variable) was the outcome in the multilevel Poisson regression (with random intercept). The individual variables included sociodemographic characteristics (age, sex, mother's level of schooling, receiving a family allowance–proxy of poverty), clinical variables (decayed teeth–DMFT≥1, and teeth affected by periodontal disease—CPI≥3), and subjective ratings (self-perception of oral health and dental anxiety).

We ran a sequence of multilevel models. The first model included only the dependent variable–i.e., the empty model (Model 1). The second and third models included the contextual variables (Model 2) and individual variables (Model 3), respectively. Next, we added the individual and contextual variables together (Model 4). We used the proportional change in variance (PCV) to assess changes in the random intercept term. According to Merlo et al. [38] the PCV represents the proportional change in the area level variance compared to the empty model (Model 1). We also used the Median Rate Ratio (MRR) in accordance to Austin et al. [39] to assess the reduction of the heterogeneity in each sequence of models. When the MRR equals 1.0, it means that there is no heterogeneity between the contexts analyzed. We tested the fitted multilevel Poisson regression model using the 2 Res log-likelihood.

## Results

A total of 2,500 students completed the questionnaires out of the 2,700 adolescents (and parents) who consented to participate in this study (92.6% rate response). The response rates were lower for the oral examinations– 67.1% to 74.0%, depending on the outcome. The reasons for missing data in the oral examination were as follows: refusal to participate in the dental examination (dental caries + periodontal) (91), took part only in the periodontal examination (171), absent on the day data collection (160), too long a delay between the survey and oral examination (400), and not filled all variables used in this manuscript (441).

The prevalence of tooth loss was 17.1% (95% CI: 15.0–19.3) and, the mean of tooth loss was 0.3 SD (0.6) per adolescent. The schools had a mean rate of participation in the National High School Examination -ENEM (2016 and, 2017) of 69.1 SD (17.2), ranging between 27.0 and 97.0. For data analysis, we used the z-score to standardize the variation in the participation between the two years (2016 and, 2017). The mean z-score for the rate of school participation in the National High School Examination among the adolescents with tooth loss was 0.0 (0.9) while it was 0.3(0.9) among adolescents with no tooth loss(p<0.001).

More participants were girls (59.0%), at the older range of adolescence (16–19 vs. 14–15) (71.3%), not receiving a family allowance (53.9%), and had a mother with more than 9 years of schooling (63.7%) (Table 1).

**Table 1:** Sample characteristics.

Variables	N (%)	School climate		Mean (SD) Tooth loss
		High/medium educational aspiration	Low educational aspiration	
**Age**	**(n = 1,237)**	**(n = 700)**	(n = **537)**	
16–19 yrs	882 (71.30)	461 (52.27)***	421 (47.73)	0.28 (0.65)*
14-15yrs	355 (28.70)	239 (67.32)	116 (32.68)	0.19 (0.54)
**Gender**	**(n = 1,237)**	**(n = 700)**	(n = **537)**	
Female	730 (59.01)	427 (58.49)	303 (41.51)	0.26 (0.64)
Male	507 (40.99)	273 (53.85)	234 (46.15)	0.24 (0.60)
**Mother's level of schooling**	**(n = 1,237)**	**(n = 700)**	(n = **537)**	
< 9 years of study	449 (36.30)	193 (42.98)***	256 (57.02)	0.36(0.74)***
≥ 9 years	788 (63.70)	507 (64.34)	281 (35.66)	0.19(0.54)
**Family allowance**	**(n = 1,237)**	**(n = 700)**	(n = **537)**	
Yes	570 (46.08)	301 (52.81)*	269 (47.19)	0.35(0.73)***
No	667 (53.92)	399 (59.82)	268 (40.18)	0.17(0.51)
**Dental anxiety**	**(n = 1,237)**	**(n = 700)**	(n = **537)**	
Yes,I`ve fear/Yes,I`ve a lot of fear	88 (7.11)	50 (56.82)	38 (43.18)	0.41(0.87)**
No/Yes, a little bit	1,149 (92.89)	650 (56.57)	499 (43.43)	0.24(0.60)
**Self-perception of oral health**	**(n = 1,237)**	**(n = 700)**	(n = **537)**	
Fair/poor	573 (46.32)	293 (51.13)***	280 (48.87)	0.36(0.73)***
Excellent/very good/good	664 (53.68)	407 (61.30)	257 (38.70)	0.16(0.50)
**Periodontitis**	**(n = 1,237)**	**(n = 700)**	(n = **537)**	
None tooth	1,182 (95.55)	672 (56.85)	510 (43.15)	0.25(0.62)
1or+	55 (4.45)	28 (50.91)	27 (49.09)	0.27(0.62)
**Decayed tooth**	**(n = 1,237)**	**(n = 700)**	(n = **537**)	
None tooth	921 (74.45)	549 (59.61)***	372 (40.39)	0.18(0.55)***
1or+ teeth	316 (25.55)	151 (47.78)	165 (52.22)	0.45(0.77)
**Type of school (26 schools)**	**(n = 1,237)**	**(n = 700)**	(n = **537**)	
Regular (17 schools)	519 (41.96)	132 (25.43)***	387 (74.57)	0.32(0.70)**
Full-time/partial time school (9 schools)	718 (58.04)	568 (79.11)	150 (20.89)	0.20(0.56)
**Social Vulnerability Index (22 neighborhoods)**	**(n = 1,237)**	**(n = 700)**	(n = **537**)	
High/very high (6 schools)	240 (19.40)	166 (69.17)***	74 (30.83)	0.28 (0.67)
Medium/low/very low (20 schools)	997 (80.60)	534 (53.56)	463 (46.44)	0.25 (0.61)

P values calculated from a T-tests for numerical variables or a Pearson’s chi-squared test for categorical variables^.^

* *p* < 0.05

** *p* < 0.01

*** *p* < 0.001

Considering the proportional change in variance (PCV), our findings showed that the reduction of heterogeneity among the schools was similar when contextual variables were added to the empty model (92.9%) versus when the individual variables were added to the empty model (92.9%). When both contextual and individual variables were added, the heterogeneity reached almost zero, i.e. the reduction was 99.9%. Hence, our findings suggested that the school academic climate contributed to explaining the tooth loss in adolescence and that when the compositional part was considered, all heterogeneity was accounted for. (Table 2)

**Table 2:** Multilevel adjustment including Social Vulnerability Index.

Parameters	Empty model (Model 1)	Random intercept, fixed effects contextual variables (Model 2)	Random intercept, fixed effects individual variables (Model 3)	Random intercept, fixed effects (individual + contextual variables) (Model 4)
Fixed part				
Individual factors				
Constant	0.26 [0.22,0.32]	0.17 [0.14,0.21]	0.07 [0.05,0.10]	0.06 [0.04,0.08]
16–19 yrs vs 14-15yrs			1.36 [1.03,1.78]	1.29 [0.98,1.70]
Female vs male			1.00 [0.79,1.25]	1.00 [0.79,1.26]
Mother's level of schooling (< 9 years of study vs ≥ 9 years)			1.49 [1.18,1.89]	1.42 [1.12,1.79]
Family allowance (yes vs no)			1.78 [1.40,2.25]	1.80 [1.42,2.28]
Dental anxiety (yes vs no)			1.36 [0.95,1.95]	1.37 [0.95,1.96]
Self-perception of oral health (Fair/poor vs Excellent/very good/good)			1.69 [1.33,2.16]	1.66 [1.30,2.11]
Periodontitis (1or+ vs 0)			0.92 [0.55,1.56]	0.90 [0.54,1.52]
Decayed tooth (1or+ vs 0)			1.90 [1.50,2.40]	1.88 [1.49,2.37]
Contextual factors (School level)				
Participation in the National High School Examination (low vs high)		1.74 [1.30,2.33]		1.42 [1.09,1.85]
Regular vs Full-time school		1.17 0.87,1.57]		1.12 [0.87,1.45]
Social Vulnerability Index by neighborhood (high vs low)		1.26 [0.93,1.69]		1.31 [1.00,1.73]
Random part				
Area level variance (Random intercept)	0.14[0.05,0.37]	0.01[0.00,2.89]	0.01[0.00,0.50]	1.01e-34
PCV^&^		-92.86%	-92.86%	-99.99%
Median Rate Ratio	MRR = 1.42	MRR = 1.10	MRR = 1.12	MRR = 1
2 Res log-likelihood	1625.76	1608.15***	1504.77***	1490.17***
Observations	1237	1237	1237	1237

Exponentiated coefficients; 95% confidence intervals in brackets

* *p* < 0.05

** *p* < 0.01

*** *p* < 0.005

PCV^&^: Proportional change in variance.

We also assessed the general contextual effect of school analyzing the MRR. In the empty model, the MRR was 1.42 suggesting that the difference in tooth loss among schools comparing similar students was 42%, i.e. a student with similar characteristics from a sorted school can be 42% more likely to have tooth loss than a student from another school. This variation in tooth loss among schools decreased in model 2 (adding contextual variables) and model 3 (adding individual variables), and in the final model 4 (both individual and contextual variables) all remaining variation between schools was accounted for, i.e. MRR = 1.

In model 3 (individual variables) and model 4 (individual and contextual variables), adolescents who presented with decayed teeth were almost twice as likely to have tooth loss, while periodontitis was not associated. Self-perception of poor oral health was also significantly correlated with tooth loss, as well as receiving a family allowance and having a mother with low schooling. In the final model, adolescents in schools with lower rate of participation in the ENEM were more likely to have tooth loss (PR 1.42, 95%CI: 1.09,1.85) while adolescents from neighborhoods with high/very high social vulnerability index and from regular vs. full-time/partial full-time school were not associated.

## Discussion

Our results corroborate our hypothesis that adolescents in a school academic climate are less likely to have tooth loss, independent of individual characteristics, such as income poverty, and low maternal educational attainment.

Our findings showed a strong association between socioeconomic factors and tooth loss, including low maternal education and receiving a family allowance. The correlation between socioeconomic disadvantage and poor oral health status is firmly established [9–11]. The common reasons that have been offered to explain this correlation include lack of financial resources to seek appropriate dental care, cultural norms that accept less-than-optimal dentition, as well as lack of health literacy. While these explanations place the responsibility on the poor, the scarcity hypothesis adds an alternative explanation, viz., the poor do not benefit from the same aspirational climate as the better off. According to Mullainathan & Shafir [12], poverty is a condition associated with uncertainty, e.g. uncertainty about the future. In that context, individuals can become more focused on the present, and fail to invest for the future. Oral health maintenance behavior is a fitting example of future orientation. An individual must engage in daily routines (brushing/flossing, avoiding sweets and cigarettes) in order to avoid dental caries and tooth loss *in the future*. A corollary is that an individual who does not perceive improving prospects would have scant motivation to invest in the future self. Brumley et al. [40] suggest that adolescents with pessimistic expectations about their college are more likely to engage in higher rates of violence. In line with this, our findings showed that oral health was affected by the school climate of low expectation about the future. Alternatively, the rate of participation in ENEM into schools might represent other unmeasured variables such as parental expectations on their children's future since this expectation might indirectly impact on the health of their offspring. Nevertheless, our finding suggests that the improvement of school academic climate might positively affect the health of our young generation [41].

The main limitation of our study is our cross-sectional design, which precludes causal inference. Our response rate to the survey was also low, although a high percentage (87.0%) of schools agreed to take part in this study. Another limitation is that 16% of our study sample were missed the oral exams. We ended the oral examinations after visiting each school at least three times to search for students who had missed earlier opportunities to receive an oral examination. By this time, three months had already passed after the administration of the survey, and we considered that the time elapsed was too long to consider using the data from the students who missed their oral examinations. Educational aspiration was assessed at the contextual level through a proxy variable–i.e., the school’s participation in the ENEM—and not evaluated at the individual level. Admittedly, educational aspirations are determined by other factors besides school educational climate, such as perceived parental support for education [42], which could affect the aspirations at the individual level. Hence it is a limitation of the current study that we did not directly measure educational aspiration in each participant, nor did we take account of other variables that might influence educational aspiration indirectly. That said, measuring and controlling for individual educational aspiration could amount to statistical over-adjustment. The point is analogous to debates about whether to control for individual SES when examining the contextual influence of neighborhood socioeconomic disadvantage on individual health outcomes. On the one hand, individual SES is a compositional confounder (prior common cause) of neighborhood poverty and individual health. At the same time, individual SES potentially lies on the pathway between neighborhood SES and individual health (because neighborhood poverty constrains individual access to educational opportunities and labor markets). The point is a very subtle one, often overlooked in multi-level studies.

Our conclusion is that school academic climate–over and above individual/school socioeconomic disadvantage–is correlated with oral health status. Our proposed mechanism of action is that a strong academic climate increases individual motivation for oral health maintenance behaviors. The reason is because a stronger academic climate sends a signal to the student that scholastic success is valued, and that excellence will be rewarded in the future by prospects of stable employment. In other words, a strong academic climate reduces uncertainty about the future, and improves the students’ capacity to plan for the future. Our argument is that when students grow and learn within a context of less uncertainty about their future, they will develop not only stronger motivation to study, but that there will be spillover benefits in other aspects of behavior–in this instance, preventive oral health maintenance behaviors.

Our findings have an important implication since tooth loss has a cumulative effect with deleterious consequences for current and future general health. A policy implication of our study is that focusing on the educational opportunities of adolescents may be a means of promoting the health of disadvantaged populations.

## Supporting information

S1 Dataset (DTA)
